# Antibodies against Platelet Factor 4 and Their Associated Pathologies: From HIT/HITT to Spontaneous HIT-Like Syndrome, to COVID-19, to VITT/TTS

**DOI:** 10.3390/antib11010007

**Published:** 2022-01-21

**Authors:** Emmanuel J. Favaloro, Leonardo Pasalic, Giuseppe Lippi

**Affiliations:** 1 Department of Haematology, Institute of Clinical Pathology and Medical Research (ICPMR), NSW Health Pathology, Westmead Hospital, Westmead, NSW 2145, Australia; Leonardo.Pasalic@health.nsw.gov.au; 2 Sydney Centres for Thrombosis and Haemostasis, Westmead Hospital, Westmead, NSW 2145, Australia; 3 Faculty of Science and Health, Charles Sturt University, Wagga Wagga, NSW 2650, Australia; 4 Westmead Clinical School, Sydney University, Westmead, NSW 2145, Australia; 5 Section of Clinical Biochemistry, University of Verona, 37129 Verona, Italy; giuseppe.lippi@univr.it

**Keywords:** platelet factor 4, antibodies, heparin-induced thrombocytopenia, vaccine-induced [immune] thrombotic thrombocytopenia, thrombotic thrombocytopenia syndrome, PF4, HIT, VITT, TTS

## Abstract

Antibodies against platelet factor 4 (PF4), a protein released from alpha-granules of activated platelets, may cause a number of pathophysiological conditions. The most commonly known is heparin-induced thrombocytopenia (HIT), which develops in a small proportion of people treated with the anticoagulant drug heparin. Notably, PF4 binds with high affinity to heparin, and in HIT, complexes of PF4/H may, in a small proportion of susceptible patients, trigger the development of anti-PF4 antibodies and subsequent platelet activation and aggregation, ultimately leading to the development of pathological thrombosis at sites of vessel occlusion. Of more modern interest, antibodies against PF4 may also arise in patients with COVID-19 (Coronavirus Disease 2019) or in patients who have been vaccinated against COVID-19, especially in recipients of adenovirus-based vaccines. For this latter group of patients, the terms VITT (vaccine-induced [immune] thrombotic thrombocytopenia) and TTS (thrombotic thrombocytopenia syndrome) have been coined. Another category associated with this pathophysiology comprises those in whom a precipitating event is not clear; this category is referred to as ‘spontaneous HIT-like syndrome’. Despite its name, it arises as an HIT-mimicking disorder but without antecedent heparin exposure. In this narrative review, we describe the development of antibodies against PF4, and associated pathophysiology, in such conditions.

## 1. Introduction

Platelet factor 4 (PF4) is a 70-amino acid protein that is stored in alpha granules of platelets and is released on platelet activation [1]. PF4 is cationic, or positively charged, and forms tetramers at physiological pH and ionic strength. Upon normal physiological platelet activation, PF4 is released as a complex with a chondroitin sulfate proteoglycan carrier and disappears rapidly from the plasma as it translocates to higher affinity heparan sulfate on endothelial cells, inhibiting local antithrombin activity and thus promoting coagulation [1]. In addition to its role in hemostasis, PF4, also known as chemokine CXCL4 (chemokine [C–X–C motif] ligand 4), has many other biological effects, which may also depend on its association with extracellular glycosaminoglycans (GAGs).

Under some conditions, pathological platelet activation can occur subsequent to the development of autoantibodies against PF4. Typically, for a particular pathophysiology, these anti-PF4 antibodies arise against PF4 in complex with negatively charged ‘species’ (i.e., molecules and polyanions), of which several candidate species exist [1]. For example, in the condition known as heparin-induced thrombocytopenia (HIT), anti-PF4 antibodies arise against PF4 in complex with heparin (i.e., anti-PF4/H antibodies are formed). These anti-PF4/H antibodies can pathologically activate platelets in a proportion of susceptible patients, leading to platelet aggregation, associated thrombocytopenia, and thrombosis at sites of vessel occlusion. In other anti-PF4 antibody pathophysiologies, the ‘associated PF4 cofactor’ may or may not be heparin and may or may not be known. In this narrative review, we describe the development of antibodies against PF4 and associated pathophysiology, inclusive of HIT and VITT (vaccine-induced [immune] thrombotic thrombocytopenia)/TTS (thrombotic thrombocytopenia syndrome).

## 2. Heparin-Induced Thrombocytopenia (HIT)

Heparin is a common parenterally administered anticoagulant [2]. This drug may be administered for one of multiple clinical indications, including thromboprophylaxis and treatment of thrombosis, potentially associated with a variety of conditions such as pulmonary embolism (PE), deep vein thrombosis (DVT), and atrial fibrillation (AF) [2]. Unfractionated heparin (UH) is a heterogeneous preparation of anionic, sulfated glycosaminoglycan polymers with molecular weights ranging from 3000 to 30,000 Da. It is a naturally occurring anticoagulant released from mast cells, binds reversibly to antithrombin, and greatly accelerates the rate at which this inhibitor inactivates the coagulation enzymes thrombin (factor IIa) and factor Xa. UH may also be administered to ensure patency of intravenous lines and circuits, such as in ECMO (Extracorporeal Membrane Oxygenation), and to prevent clotting in surgery, especially cardiac and arterial surgery, or during dialysis. Low-molecular-weight heparin (LMWH) represents a fractionated product with a lower average molecular weight (about 4.5 kDa) than UH (~15 kDa). UH can be administered by continuous intravenous infusion or by subcutaneous injection, whilst LMWH is typically administered by subcutaneous injection.

**Figure 1:**
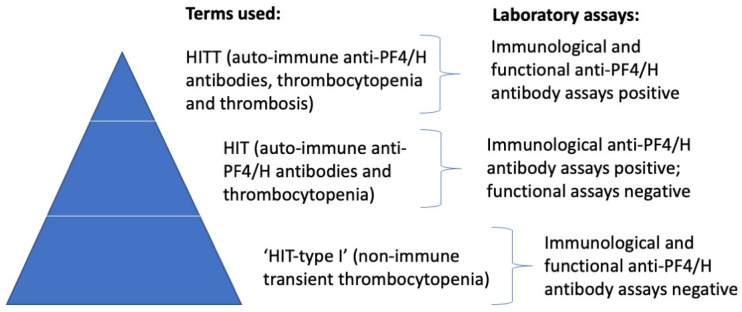
The HIT pyramid. Most patients with thrombocytopenia after initiation of heparin therapy will reflect a non-immune and transient platelet drop; these patients will be negative for anti-PF4 antibodies using immunological assays and functional platelet activation assays. However, some patients with thrombocytopenia after initiation of heparin therapy will reflect an immune-mediated platelet drop due to the production of anti-PF4 antibodies and the clearance of platelets (‘HIT’). These patients will be positive for anti-PF4 antibodies using immunological assays but negative by functional platelet activation assays. Only a small proportion of patients (at the ‘top’ of the pyramid) will have HITT, being HIT patients with resultant thrombosis, due to platelet activation, aggregation, and thrombotic vessel occlusion. These patients will be positive for anti-PF4 antibodies using immunological assays, as well as by functional platelet activation assays.

In some patients, administration of heparin is associated with a reduction in platelet count [3]. In most of these patients, this is transient, and platelet counts return to normal. This is sometimes called HIT type I and results from a direct effect of heparin on platelets (i.e., it represents a non-immune event). In a small subset of patients treated with heparin, antibodies can develop against the PF4/H complex and cause an immune-mediated clearance of platelets. These antibodies can be detected by a variety of assays, as further highlighted below, but will not lead to significant pathophysiology in most patients. However, in a small subset of patients treated with heparin, at the top of the HIT-pyramid (Figure 1), the PF4/H antibodies that are formed can cause an immune-mediated activation of platelets. This activation event can be assessed by laboratory testing by using a range of functional assays, including serotonin-release assays (SRA) and heparin-induced platelet aggregation (HIPA) assays. In vivo, these platelet-activating PF4/H antibodies will lead to platelet aggregation and also to thrombosis, which some workers term HITT (heparin-induced thrombotic thrombocytopenia). The risk of HITT is higher in females than in males, magnified by the use of UH compared to LMWH, and more frequent in some clinical situations (e.g., post-surgery, patient on ECMO, etc.).

The identification or exclusion of HIT (or HITT) requires a process that entails a careful clinical assessment, especially focused on the presence of a thrombosis, the proximity to use of heparin, as well as laboratory testing [3,4,5]. The clinical evaluation is often facilitated by means of a scoring system, of which the so-called “4Ts” is the most common [6,7,8]. The 4Ts involves giving ‘points’ according to: (1) presence of Thrombocytopenia (or >30% fall in platelet count), (2) Timing of platelet count fall (typically between 5 and 10 days after initiation of heparin therapy), (3) presence of Thrombosis or other sequelae, (4) ‘absence’ of other causes of Thrombocytopenia. The maximum score is 8, and scores of 0–3, 4–5, and 6–8 respectively identify ‘low’, ‘intermediate’, and ‘high’ probability of HIT (or HITT).

**Figure 2:**
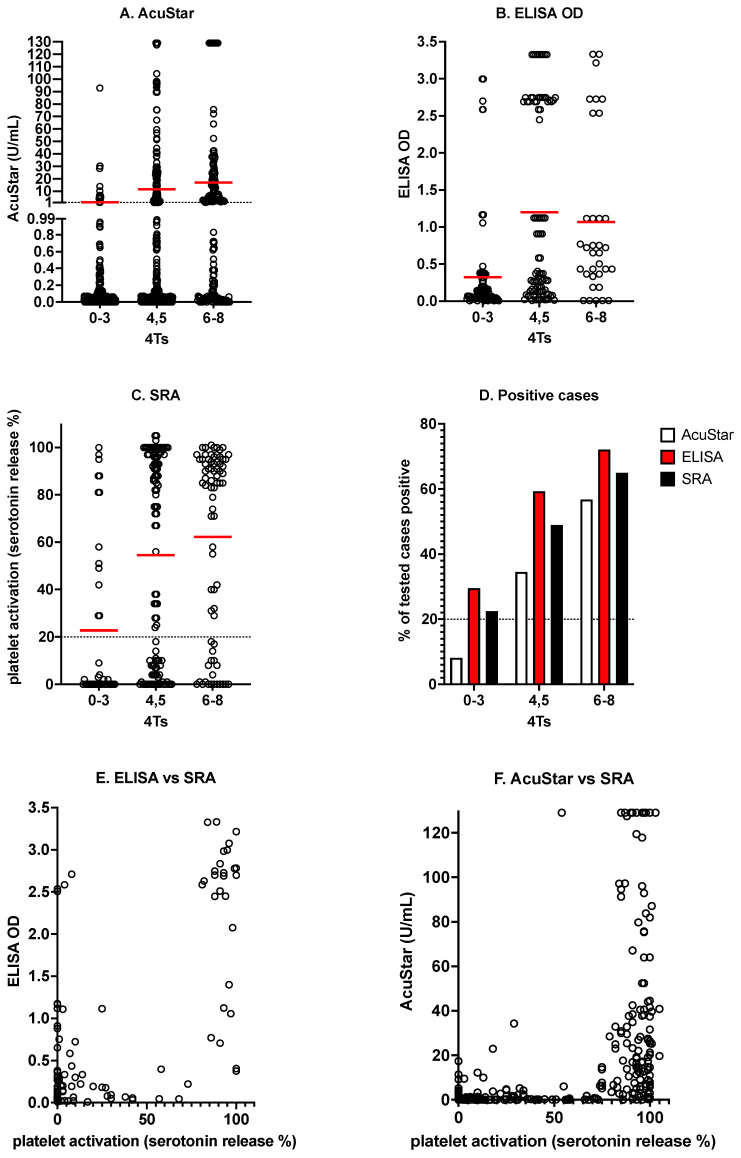
Some examples of testing for HIT according to 4T score (4Ts) and reactivity profiles. The higher the 4Ts, the more likely the presence of PF4/H antibodies. Thus, the higher the 4Ts, the higher the expected result in the AcuStar chemiluminescence assay (**A**), the higher the expected result by ELISA OD (optical density; (**B**)), and the greater the expected level of platelet activation by SRA (**C**) (the red dash in each score group indicates the average value for the dataset). The higher the 4Ts, the higher the expected proportion of tested cases being positive by each assay (**D**). The higher the titer of immunologically detected PF4/H antibodies, the more likely the positivity in functional platelet activation assays (**E**,**F**). Data shown here are historical multicenter data, including from the Westmead laboratory, as partly previously reported [9,10]. OD, optical density.

In addition to laboratory testing for the presence of thrombocytopenia, the presence of antibodies against PF4/H is initially investigated by immunological testing, for which a variety of assays have now become available [3,4,5]. These include ELISAs (enzyme-linked immunosorbent assays) and various rapid assays including lateral flow, chemiluminescence, latex and particle gel immunoassays. All such techniques are sensitive to detecting PF4/H (or HIT) antibodies but have variable specificity for pathophysiological HIT (or HITT). Among these assays, the chemiluminescence-based methods probably have the highest specificity for HITT [9,10,11,12]. Once PF4/H (or HIT) antibodies have been detected by these immunoassays, the presence of such antibodies is proved, but functional methods based on platelet activation and/or aggregation should be performed to identify if these antibodies are pathologically able to activate platelets [3,4,5,9,10]. The most common historical laboratory techniques to assess platelet activation include SRA and HIPA, but given these assays are complex or not widely available, alternate or complementary methods are emerging, such as those based on flow cytometry [13,14,15]. To help illustrate this process, Figure 2 shows some examples of results of testing by several immunological assays, as well as for SRA, for various grades of 4Ts, using Australian test data, including those from the laboratory managed by two of the authors (Westmead Hospital).

**Figure 3:**
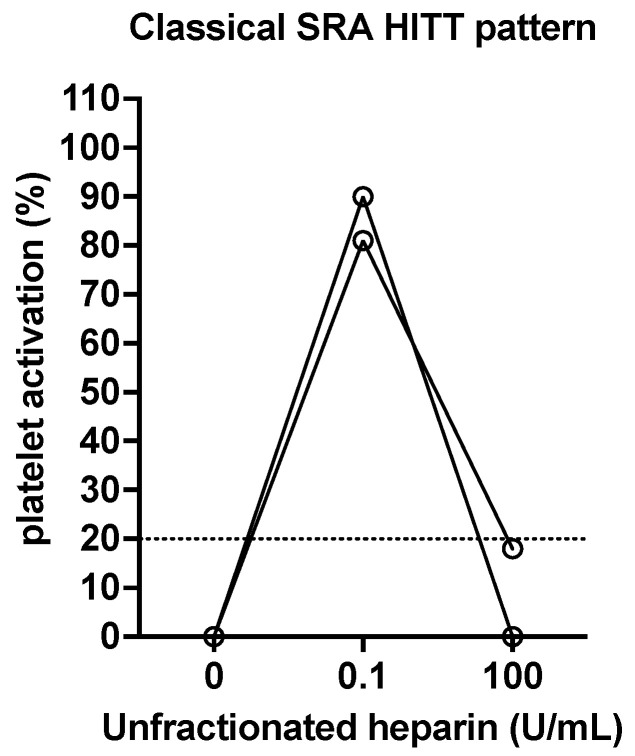
Two examples of a classical pattern expected for HITT positive cases assessed by SRA. In the absence of added heparin, no platelet activation should occur. In the presence of added heparin at a therapeutic level (0.1 U/mL final concentration in these examples), platelet activation, measured as serotonin release, occurs. In the presence of added heparin at a supra-therapeutic level (100 U/mL final concentration in these examples), platelet activation, measured as serotonin release, is inhibited (i.e., no release occurs). Occasionally, platelet activation may also occur in the absence of added therapeutic heparin level, due to the presence of either strong antibodies or heparin in the patient’s plasma. Occasionally, again due to the presence of strong antibodies, platelet activation may not fully normalize in the presence of an administered supratherapeutic heparin level.

In functional assays such as SRA (and HIPA), one can also assess the effect of added heparin on platelet activation/aggregation. In these ‘modified’ assays, in the absence of added heparin, the tests may or may not identify platelet activation/aggregation. In the presence of a therapeutic level of heparin (e.g., 0.1 or 0.5 U/mL), the presence of platelet activation/aggregation is taken as an indication of pathological PF4/H antibodies (i.e., suggestive of HITT). Indeed, activation/aggregation at a therapeutic level of heparin is often greater than that in the absence of heparin. Finally, a high dose (supra-therapeutic, e.g., 10 to 100 U/mL) of heparin can be used to show inhibition of platelet activation/aggregation (i.e., confirmatory of HITT). Examples of a classical pattern by SRA are shown in Figure 3, using data from patients from the Westmead Hospital laboratory.

## 3. Spontaneous HIT-Like Syndromes

It is also possible to develop anti-PF4 antibodies without evident prior or proximate exposure to heparin [16,17,18]. As these do not appear to arise due to heparin exposure, they are not really anti-PF4/H antibodies but may represent PF4 in an alternate complex, which we will designate here as “anti-PF4/X antibodies”, where “X” is an anionic species, but typically not heparin. Of interest, the same immunological assays used to identify anti-PF4/H antibodies in HIT (i.e., ELISA or rapid assays) can also be used to identify these anti-PF4/X antibodies, although there may be differences in both sensitivity and specificity, in part because the laboratory assays have been designed to preferentially measure anti-PF4/H antibodies (i.e., antibodies against PF4 in complex with other anionic species will be less reactive). The term ‘spontaneous HIT’ is sometimes used, but this term is perhaps a misnomer, given heparin does not appear to be involved (so, maintaining the above convention, the term ‘XIT’ could be considered more appropriate). Irrespective of this, the term ‘spontaneous HIT-like syndrome’ would perhaps be an appropriate option.

An excellent review on this syndrome has recently been published [16]; therefore, we will only provide a brief overview here. First described in 2008, two subtypes of this syndrome have been identified: (a) surgical (post-orthopedic, especially post-total knee arthroplasty) and (b) medical (usually post-infectious). A wide variety of polyanions may form complexes with PF4, and for spontaneous HIT-like syndromes, these potentially include bacteria (specifically lipid A in bacterial surfaces) or certain nucleic acids such as DNA and RNA.

In these patients, anti-PF4 ELISA antibody assays are positive, and to some extent, different from HIT, SRA testing will typically show platelet activation in the absence of added heparin, which would not expectedly increase by the addition of a therapeutic heparin level. Finally, a high dose (supra-therapeutic) of heparin can be used to show inhibition of platelet activation/aggregation. These ‘spontaneous HIT-like syndromes’ represent rare events and are not widely reported, or else they may be confused with heparin-related HIT. In the recent noted review [16], a total of 27 reports were identified as related to recent infection or knee surgery.

## 4. Anti-PF4 Antibodies in COVID-19 Patients

COVID-19 (Coronavirus Disease 2019) is a now well-recognized pandemic caused by infection with the virus SARS-CoV-2 (Severe Acute Respiratory Syndrome Coronavirus 2). COVID-19 is a clearly prothrombotic disorder that involves multiple hemostasis pathways of interest, including platelet activation [19]. However, thrombosis in COVID-19 patients is multi-factorial, and platelets only play a part in a larger coagulopathic process. Unlike in HIT patients, platelet counts in COVID-19 patients are not usually very low, and so these patients are considered only mildly thrombocytopenic. In COVID-19, multiple hemostatic pathways can be affected, including primary hemostasis (platelets and von Willebrand factor [VWF]), secondary hemostasis (‘coagulation’), and fibrinolysis. Moreover, anti-PF4 antibodies do not arise in the majority of COVID-19 patients [20] and so cannot be considered a major driver of COVID-19-associated coagulopathy.

Nevertheless, of relevance to the current review, beside direct platelet activation resulting from direct interaction of SARS-CoV-2 spike protein with platelet receptors [15], ‘HIT-like’ events may occur in a small proportion of patients with COVID-19, and there have been several reports of anti-PF4 antibodies in COVID-19 patients, as recently reviewed by some of us [20]. In some cases, these were identified as involving heparin (i.e., anti-PF4/H antibodies were identified); however, in other cases, they did not involve prior heparin exposure (i.e., they were not anti-PF4/H antibodies, and so can be considered anti-PF4/X antibodies). Indeed, in some reports, the addition of therapeutic heparin levels in an assay can be shown to decrease antibody detection by immunological assays or inhibit platelet activation in functional assays, further confirming that these are not anti-PF4/H antibodies. Nevertheless, these events cannot really be considered as a form of ‘spontaneous HIT-like syndrome’, since SARS-CoV-2 is a likely trigger in at least some of these patients. Nevertheless, they do likely represent an analogous entity to the HIT-like syndromes mentioned in the preceding section, albeit associated with a particular viral infection, being SARS-CoV-2 [16]. Whether the anionic species associated with PF4 (i.e., the ‘X’ in PF4/X) is part of the virus, part of an associated co-bacterial infection, or simply arises due to platelet activation and consequent complex formation is still unknown.

In summary, a small proportion of COVID-19 patients may have anti-PF4/X antibodies, only a fraction of which can be identified as anti-PF4/H antibodies, with the remainder representing antibodies against PF4, potentially in complex with an as yet unknown ‘anionic species’ (‘X’) [20,21,22]. From the laboratory testing perspective, these will be anti-PF4 ELISA antibody-positive by immunological assessment, and SRA testing may show platelet activation in either presence or absence of added therapeutic heparin, depending on whether antibody development was due to heparin exposure (PF4/H complexes) or not (PF4/X complexes). In either case, a high dose (supra-therapeutic) of heparin should show inhibition of platelet activation/aggregation.

## 5. Anti-PF4 Antibodies in VITT/TTS

The COVID-19 pandemic has led to the rapid production and deployment of a large number of COVID-19 vaccines [23,24]. Notably, some minor adverse reactions may be anticipated for any vaccination program. Unfortunately, occasional rare and potentially fatal adverse events may also arise. One such event appears to arise in a small proportion of individuals vaccinated with COVID-19 adenovirus-based vaccines. Termed VITT (for vaccine-induced [immune] thrombotic thrombocytopenia) by the workers who first reported on the associated pathophysiological events [25,26,27], the term TTS (for thrombotic thrombocytopenia syndrome) may preferentially be used by government reporting agencies (for example, the FDA [Food and Drug Administration] in the USA, the EMA [European Medicines Agency] in Europe, and the TGA [Therapeutic Good Administration] in Australia) [23]. VITT and TTS after COVID-19 vaccine use essentially represent the ‘same’ condition, albeit that the specific case definition used to define VITT or TTS may result in the recognition of different patient cohorts. Even within the entity described as VITT, different patient cohorts may be identified, according to the diagnostic pathway used [28]. In a recent review, some half-dozen diagnostic pathways were identified as being recommended by various expert groups [28]. Whilst the pathways in general aimed to accurately identify VITT patients, differences in the approach could lead to inclusion or exclusion of some cases relative to a different diagnostic approach. For example, some diagnostic pathways restricted the inclusion of cases up to 28 days post-vaccine exposure, whereas others captured cases up to 42 days post-exposure [28]. Some diagnostic pathways placed stronger emphasis on D-dimer measurements than others, and some diagnostic pathways restricted case capture only to patients with thrombocytopenia, whereas some pathways included case capture for patients without thrombocytopenia but with a substantive fall in platelet counts, akin to 4Ts in HIT assessment.

**Figure 4:**
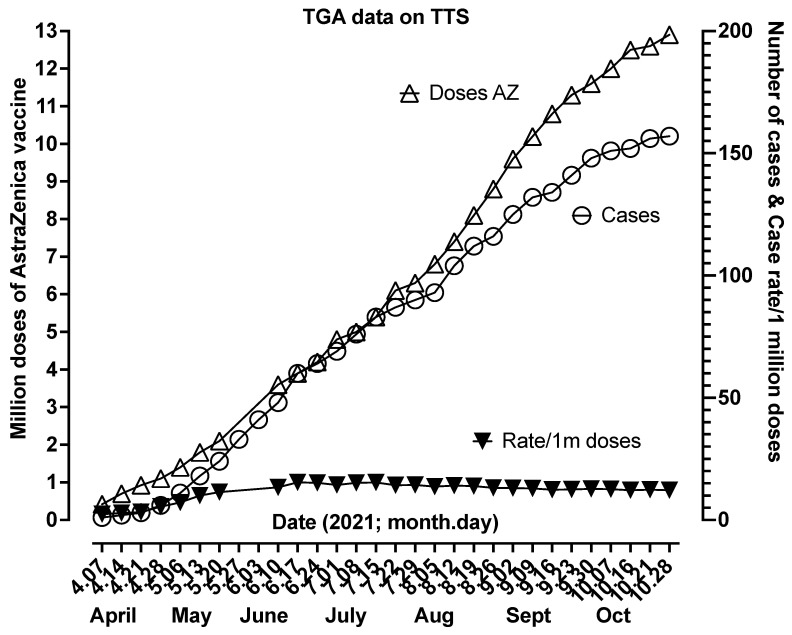
Cases of TTS as reported weekly by the Australian TGA from the first case reported at the beginning of April 2021 until the end of October 2021. The figure shows the cumulative number of AstraZeneca (AZ) doses administered, the cumulative number of TTS cases, and the TTS rate per million AZ doses. From an initial slow case attainment, the rate seemed to stabilize from June to October at around 13–15/million doses. Please see [31] for additional details.

VITT/TTS also has a recognized prevalence, albeit potentially different according to the vaccine with which it occurs. For example, for the AstraZeneca vaccine (also known as ChAdOx1 nCoV-19, AZD1222, Vaxzevria), the prevalence is around 1 in 80,000 doses or between 10 and 15 cases per million doses (Figure 4). For the Janssen (Johnson & Johnson) vaccine (alternatively known as Ad26.COV2.S or JNJ-78436735), the prevalence seems to be lower, perhaps 1 in 500,000 doses, or ~2 cases/million doses [23]. The prevalence of VITT/TTS will also differ according to the case definition and the diagnostic pathway chosen, as further outlined above, of which there are many [28]. For example, a total of only 58 cases of TTS worldwide were identified by one recent systematic review as of 23 August 2021, as based on WHO criteria for TTS identification [29]. In contrast, a separate review performed by one of us [30] identified at least 83 cases of VITT worldwide as of a much earlier date, i.e., 27 May 2021, and based on clinical presentations and results of laboratory tests. Thus, the systematic review would significantly underestimate the number of TTS cases worldwide, which currently stand at >150 for Australia alone, according to a recent TGA report (Figure 4).

**Figure 5:**
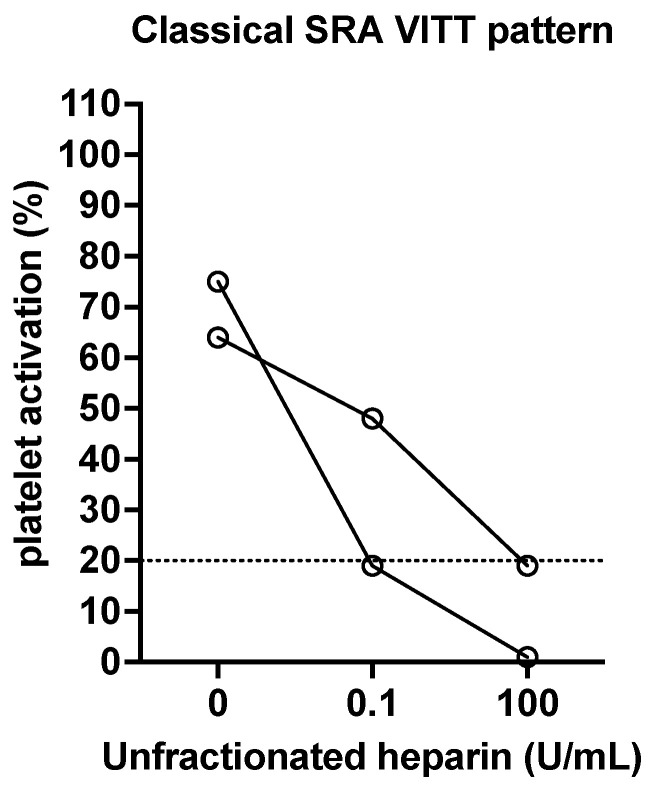
Two examples of a classical pattern expected for VITT-positive (Westmead Hospital) cases assessed by SRA. Platelet activation should occur in the absence of added heparin. In the presence of added heparin at a therapeutic level (0.1 U/mL final concentration in these examples; 0.5 U/mL may lead to even greater inhibition), platelet activation, measured as serotonin release, may be inhibited. In the presence of added heparin at a supra-therapeutic level (100 U/mL final concentration in these examples), platelet activation, measured as serotonin release, is further inhibited (i.e., very little or no release occurs). Occasionally, however, platelet activation may also occur in the presence of added therapeutic heparin, thereby challenging the discrimination between VITT and HITT.

Of major relevance to the current review, VITT/TTS is also characterized by the presence of anti-PF4 antibodies, or more likely anti-PF4/X antibodies, with X being an unknown anionic ‘species’ at present (perhaps, heparan sulfate proteoglycans or vaccine components such as adenovirus-derived hexon) [32,33]. Again, these anti-PF4 antibodies can be detected by immunological assays, but unlike in HIT, not all such assays can identify these antibodies with sufficient diagnostic sensitivity. Indeed, only the ELISA-based techniques can consistently identify anti-PF4 (or anti-PF4/X) antibodies in VITT/TTS, with all other immunological methods, including rapid assays, either not detecting the antibodies or only detecting these in a minor proportion of patients (i.e., typically below 30%) [21]. This tends to support the concept that the ‘X’ in anti-PF4/X for VITT/TTS is not heparin. Perhaps also interesting here is that the in vitro use of heparin in laboratory testing tends to reduce the level of detected antibodies in immunological assays for the majority of VITT patients and tends to inhibit platelet activation in functional assays, essentially proving that the species in complex with PF4 (at least, in the majority of patients) is not heparin. An example of this for two patients from the Westmead Hospital laboratory is provided in Figure 5. However, therapeutic heparin does not inhibit platelet activation in all VITT patients [34,35], and so this does not provide an infallible distinction from HITT.

## 6. Discussion

In this review, we have provided some insights into anti-PF4 antibodies and their presence in certain pathophysiological states. It is interesting that these pathophysiological states can arise in normal individuals, either when under treatment with heparin or as a rare outcome of COVID-19 or vaccination against COVID-19. Alternatively, the antibodies can arise as part of another pathological process, such as in the presence of other infections, after certain surgeries, or else from otherwise unknown causes (‘spontaneous’). There are both similarities and differences in these presentations and in laboratory test results. For HIT, the expected presentation of 5–10 days post-heparin initiation is much tighter that that expected for VITT, which may be of 5–42 days, albeit having a similar minimum presentation period of ~5 days [36]. For HIT, all immunological anti-PF4/H antibody assays can detect the anti-PF4/H antibodies with high sensitivity, albeit with differing specificity, with the rapid chemiluminescence assay perhaps showing highest specificity [10,11,12]. For VITT, only ELISA-based immunological assays are consistently sensitive to anti-PF4/X antibodies, with rapid assays, otherwise sensitive to anti-PF4/H antibodies in HIT, being mostly negative in VITT [23,28,37]. Moreover, the addition of therapeutic levels of heparin augments the detection of anti-PF4/H antibodies in HIT/HITT, by both immunological and functional assays, whereas for VITT, therapeutic levels of heparin may reduce the detection of anti-PF4/X antibodies by immunological (ELISA) testing and inhibit platelet activation in functional assays [30,35]. This is probably due to the fact that anti-PF4 antibodies in HIT vs. VITT target different epitopes on PF4 and thus may compete for PF4 binding, as recently shown by Huynh and colleagues [38]. These similarities and differences have important diagnostic implications, as well as enabling the differentiation of the disorders to some extent. They may also provide pointers for their differential management. For example, heparin is clearly contraindicated in HITT but not always so clearly contraindicated in VITT, although most experts would be cautious about its use in these patients. Importantly, some cases of VITT do not show heparin inhibition, and so this may point to heterogeneity in VITT anti-PF4 antibodies, where perhaps the ‘X’ in PF4/X does not always corresponds exactly to the same anionic entity in all patients. Table 1 provides a summary of the expected laboratory features of the different anti-PF4 antibody conditions.

## 7. Conclusions

**Figure 6:**
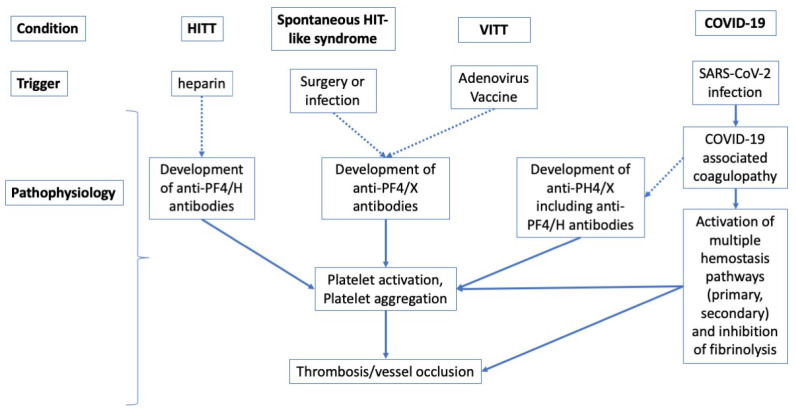
Navigating the differences and similarities of anti-PF4 antibody development in the different conditions considered in this review. ‘H’ in anti-PF4/H refers to heparin as the PF4 cofactor; ‘X’ in anti-PF4/X refers to a PF4 cofactor that may or may not be heparin. All conditions can lead to the development of anti-PF4 antibodies, which are anti-PF4/H in HITT and in some cases of COVID-19, and which are probably not anti-PF4/H in ‘spontaneous HIT-like syndrome’, in VITT and in a minority of cases of COVID-19. In COVID-19 patients, a generalized infection-associated coagulopathy may develop, in which only rarely will development of anti-PF4/X antibodies occur. Most cases of thrombosis in COVID-19 patients are thus unrelated to the development of anti-PF4/X antibodies.

Several lines of evidence now attest that autoantibodies against PF4 may arise under a large number of specific conditions, with several additional risk factors or associations, including disease, infection, and surgery, and as noted by a wide variety of researchers [3,16,20,23,30,39,40,41,42,43,44,45,46,47,48,49,50,51,52,53]. In a proportion of patients, those with platelet-activating anti-PF4 antibodies, such conditions and risk factors, together with platelet activation and aggregation, consequently expose them to a substantially enhanced risk of thrombosis. These anti-PF4 antibodies typically develop against PF4 complexed with some negatively charged molecules, such as heparin (e.g., in HITT) or potentially other polyanionic species, which still remain largely unidentified (e.g., in VITT or spontaneous HIT-like syndromes). Although the mechanism(s) underlying their generation probably share some similar characteristics (Figure 6), they seem to have heterogeneous composition and molecular targets depending on the clinical conditions in which they may arise. Therefore, thoughtful interrogation of clinical history (i.e., heparin exposure, SARS-CoV-2 infection, administration of COVID-19 vaccines, and so forth) and physical assessment (i.e., type and site of thrombosis) are essential, since they will guide the diagnostic reasoning (i.e., the choice of the laboratory assay(s)) and the consequent clinical decision making (i.e., type and duration of anticoagulant or anti-thrombotic treatment).

Some additional insights into the differences between the antibodies formed in different pathologies have recently emerged. Singh et al. [35], in an interesting study, albeit including only a small number of cases, showed that antibodies from HITT patients tended to be polyclonal, whereas those from patients with VITT and from one case of ‘spontaneous HIT’ were oligoclonal. Moreover, immunological antibody detection by ELISA for VITT cases was the most accurate when using noncomplexed PF4, followed by PF4 in complex with polyvinyl sulfonate (PVS), and the least accurate with PF4 in complex with heparin (PF4/H).

## Figures and Tables

**Table 1:** Summary table. Expected laboratory test patterns in different types of anti-PF4 antibody syndromes ^1^.

Anti-PF4 Antibody Syndrome	Immunological Assays	Functional Assays
Heparin-induced thrombocytopenia (HIT) (with thrombosis; HITT)	All anti-PF4 antibody assays expected to be positive	Expected to be positive in those with HITT (particularly in the presence of therapeutic heparin (e.g., 0.1 or 0.5 U/mL)), but not in those without pathological HIT. Negative in the presence of supratherapeutic heparin (e.g., 10 or 100 U/mL)
Spontaneous HIT-like syndrome	ELISA-based anti-PF4 antibody assays expected to be positive; insufficient information on other anti-PF4 antibody assays	Expected to be positive in the absence of therapeutic heparin. Negative in the presence of supratherapeutic heparin.
COVID-19	ELISA-based anti-PF4 antibody assays expected to be positive; other anti-PF4 antibody assays also in general will be positive	Expected to be positive, but different findings may be evident in the presence or absence of therapeutic heparin, depending on the ‘trigger’ for antibody development. All cases should be negative in the presence of supratherapeutic heparin.
VITT/TTS	ELISA-based anti-PF4 antibody assays expected to be positive; other anti-PF4 antibody assays, including rapid assays, expected to be generally negative	Expected to be positive in the absence of heparin. Most will show some inhibition in the presence of therapeutic heparin. All cases should be negative in the presence of supratherapeutic heparin.

^1^ There may be some heterogeneity in patterns on a case-by-case basis. Sometimes, it is not entirely clear what entity has arisen. For example, it is possible for a patient to have been given a COVID-19 vaccine, be admitted to hospital, have heparin administered, and then have a fall in platelet count and a thrombosis. Here, either HITT and/or VITT may be present.

## Data Availability

Not applicable. This review does not report any data not otherwise available in the public domain.
